# Experimental models of hepatic injury in Wilson disease: stage-specific mechanisms and translational applications

**DOI:** 10.3389/fimmu.2026.1930939

**Published:** 2026-09-28

**Authors:** Ting Cheng, Nannan Qian, Hu Xi, Wenjie Hao, Yue Yang, Wenming Yang

**Affiliations:** 1 The First Affiliated Hospital of Anhui University of Chinese Medicine, Hefei, China; 2 Brain Disease Research Institute, Anhui University of Chinese Medicine, Hefei, China; 3 Anhui University of Chinese Medicine, Hefei, China; 4 Key Laboratory of XinAn Medicine, Ministry of Education, Hefei, China

**Keywords:** ATP7B, experimental models, hepatic injury, liver fibrosis, Wilson disease

## Abstract

**Background:**

Wilson disease (WD) is an autosomal recessive disorder of copper metabolism caused by pathogenic variants in ATP7B, with the liver being one of the earliest and most prominently affected organs. ATP7B dysfunction impairs copper incorporation into ceruloplasmin and biliary copper excretion, resulting in progressive hepatic copper accumulation and a cascade of pathological responses, including proteostasis disturbance, mitochondrial dysfunction, oxidative stress, metabolic remodeling, inflammation, and fibrogenesis. Given the dynamic progression of WD-associated liver injury from early copper dysregulation to chronic hepatitis, fibrosis, cirrhosis, and acute liver failure-like decompensation, appropriate experimental model selection is essential for mechanistic studies and therapeutic development.

**Methods:**

We conducted a structured narrative review of *in vivo* and *in vitro* models of WD-associated hepatic injury, with searches updated through August 2026, and evaluated each model according to genetic fidelity, disease-stage representation, dominant mechanism, tissue complexity, and translational objective. We then developed a stage-, mechanism-, and objective-oriented model-selection framework.

**Results:**

Different WD models recapitulate distinct stages and features of hepatic injury. Severe Atp7b-deficient rat models, including the LEC rat, are particularly suitable for investigating severe liver injury, mitochondrial copper toxicity, and rapid decoppering interventions. tx-j mice and early-stage Atp7b−/− mice effectively model early hepatic copper accumulation, metabolic remodeling, and epigenetic alterations. Atp7b−/− mice, tx mice, LEC survivors, and Labrador retriever copper-associated hepatitis models are valuable for studying chronic copper hepatopathy, inflammation, and fibrosis. Variant-specific models are most informative for defining mutation-level defects in ATP7B folding, trafficking, stability, or transport and for testing mutation-compatible interventions; they should not be interpreted as establishing deterministic clinical genotype–phenotype relationships. Direct ATP7B-deficient models and copper-overload reference models are treated as distinct experimental categories throughout this Review. For fibrosis research, simple copper-exposed hepatocyte or hepatic stellate cell systems are insufficient to reproduce disease complexity.

**Conclusions:**

No single experimental model adequately captures the full spectrum of WD-associated hepatic injury. Model selection should be guided by the experimental question across disease stage, mechanistic hypothesis, genetic fidelity, tissue complexity, and translational objective. The most robust strategy is a tiered model-selection and validation pipeline in which genetic fidelity, tissue complexity, and therapeutic endpoint are increased as a finding moves from mechanistic discovery toward preclinical translation. WD-specific multicellular organoid and liver-on-chip systems remain a major unmet methodological need.

## Introduction

Wilson disease (WD) is an autosomal recessive disorder of copper metabolism caused by pathogenic variants in the ATP7B gene, which encodes a copper-transporting P-type ATPase that plays a central role in hepatic copper homeostasis (1–3). Under physiological conditions, ATP7B facilitates copper incorporation into ceruloplasmin within the trans-Golgi network and mediates biliary copper excretion when intracellular copper levels increase (4, 5). Loss of ATP7B function disrupts both processes, leading to progressive hepatic copper retention and subsequent injury to multiple cellular systems. As copper accumulates, hepatocytes undergo a broad spectrum of pathological alterations, including proteostasis imbalance, mitochondrial dysfunction, oxidative stress, autophagy dysregulation, lipid metabolic remodeling, one-carbon metabolism disturbances, inflammatory activation, and fibrogenesis (6–11).

The liver is typically the first organ affected in WD and remains one of the major determinants of clinical outcome (1, 2). Hepatic manifestations are highly heterogeneous, ranging from asymptomatic biochemical abnormalities and steatosis to chronic hepatitis, advanced fibrosis, cirrhosis, acute liver failure-like presentations, and, in some cases, hepatocellular carcinoma (6–8). This heterogeneity suggests that WD-associated liver disease should not be viewed as a single pathological entity but rather as a dynamic continuum extending from early copper dysregulation to progressive tissue remodeling and hepatic decompensation. Different biological processes dominate different stages of disease progression, and the mechanisms driving early adaptive responses may differ substantially from those involved in advanced liver injury.

Although copper chelators, zinc therapy, dietary copper restriction, and liver transplantation have substantially improved patient prognosis, several fundamental questions remain unresolved. These include how hepatocellular copper retention evolves into irreversible liver injury, how distinct ATP7B variants influence hepatic phenotypes, how mitochondrial dysfunction, proteostasis defects, inflammation, and fibrosis are interconnected during disease progression, and which experimental systems are most appropriate for evaluating emerging therapeutic approaches such as gene replacement, mutation-specific rescue, rapid decoppering strategies, and antifibrotic interventions (6, 12–17). Because access to human liver tissue is limited and frequently confounded by disease stage, treatment history, and genetic heterogeneity, experimental models remain indispensable for mechanistic studies and preclinical therapeutic development.

Over the past decades, a wide spectrum of WD-related experimental models has been established, including spontaneous animal models, genetically engineered Atp7b-deficient animals, variant-specific knock-in models, canine comparative models, ATP7B-deficient hepatocyte systems, patient-derived induced pluripotent stem cell (iPSC)-derived hepatocyte-like cells (HLCs), hepatic stellate cells (HSCs), liver sinusoidal endothelial cells (LSECs), organoids, and multicellular three-dimensional (3D) platforms (18–28). However, these models are often discussed collectively despite representing fundamentally different biological contexts. Individual systems vary substantially in genetic background, copper burden, disease severity, progression kinetics, cellular composition, and translational applicability (15–17). For example, LEC rats and Atp7b-deficient rat systems predominantly reproduce severe hepatic injury and mitochondrial copper toxicity (12, 13, 29), whereas tx-j mice are particularly useful for studying early metabolic and epigenetic alterations. Atp7b−/− mice have become standard platforms for chronic disease progression and therapeutic testing (30–32), while mutation-specific models such as ATP7B-H1069Q-, R778L-, or R780L-based systems enable investigation of variant-dependent molecular defects and mutation-compatible therapeutic strategies.

An additional challenge in the field is the frequent conflation of ATP7B-deficient WD models with exogenous copper-overload systems. Direct WD models carry ATP7B mutations, deletions, or patient-derived ATP7B-deficient backgrounds and therefore reproduce the primary genetic defect underlying the disease (21–23). In contrast, wild-type hepatocytes or stellate cells exposed to exogenous copper mainly reflect cellular responses to copper excess and adaptive copper handling rather than ATP7B-deficiency-driven pathogenesis (18, 24). Throughout this Review, we therefore use “copper-toxicity reference model” for ATP7B-intact systems challenged with exogenous copper and reserve “direct WD model” for ATP7B-deficient or disease-variant backgrounds.

The same issue applies to fibrosis research. Hepatic fibrosis in WD is not merely a consequence of copper accumulation within hepatocytes but rather the result of complex interactions among injured hepatocytes, activated HSCs, dysfunctional LSECs, inflammatory cells, and extracellular matrix remodeling. Consequently, monoculture systems based solely on hepatocytes or stellate cells provide only a partial representation of the fibrogenic process. Recent advances in co-culture systems, patient-derived cellular platforms, organoids, and liver-on-chip technologies have created new opportunities to model multicellular interactions involved in copper-associated fibrogenesis (25–28, 33, 34). However, general liver organoid or liver-on-chip technologies are not considered WD-specific unless they incorporate an ATP7B-deficient or disease-variant hepatocyte compartment.

Despite the growing diversity of available models, existing reviews have generally categorized WD systems according to species, genetic background, or experimental platform rather than according to the biological processes and disease stages they represent (6, 16, 17). The novelty of the present framework is the addition of a decision layer that links five variables—disease stage, dominant mechanism, genetic fidelity, tissue complexity, and translational objective—to model choice. This shifts the practical question from “Which WD model is best?” to “Which model is fit for this biological or therapeutic question, and what orthogonal model is required for validation?” **Figure 1** provides a concise model landscape and decision workflow, whereas **Table 1** compares preferred and confirmatory models for overlapping applications.

**Figure 1:**
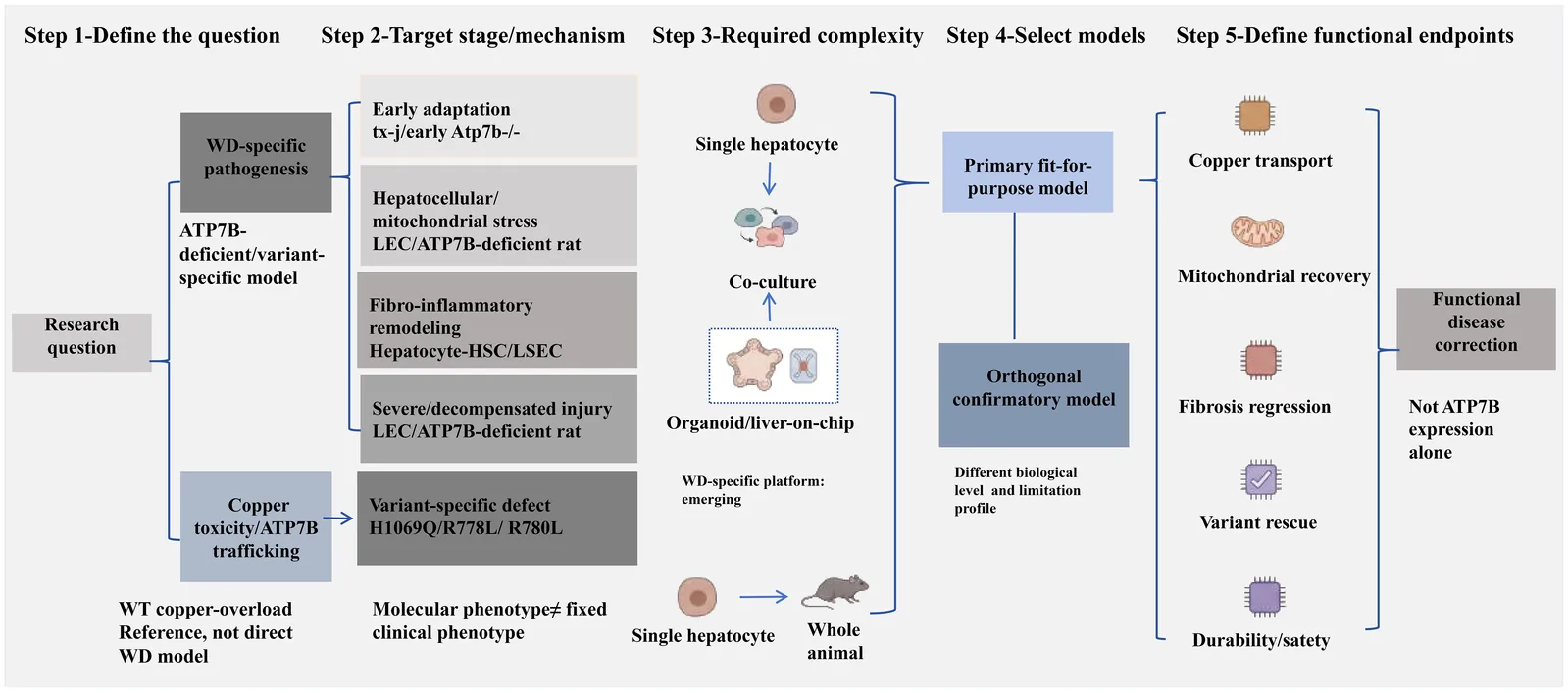
Stage-, mechanism-, and objective-oriented decision framework for selecting Wilson disease experimental models. Model selection follows five steps. Step 1, define the question: WD-specific pathogenesis requires ATP7B-deficient or variant-specific models, whereas ATP7B-intact copper-overload systems primarily model general copper toxicity or ATP7B trafficking. Step 2, target the stage or mechanism: match models to early adaptation, cellular/mitochondrial stress, fibro-inflammatory remodeling, advanced injury, or variant-specific defects. Step 3, define biological complexity: select hepatocytes, multicellular co-cultures, organoid/liver-on-chip systems, or whole animals according to the question. Step 4, select models: where appropriate, complement a primary model with an orthogonal confirmatory model. Step 5, define functional endpoints: assess copper transport, mitochondrial recovery, fibrosis regression, variant-specific rescue, durability, and safety. Successful disease correction should be defined by restoration of disease-relevant function rather than ATP7B expression alone.

**Table 1:** Context-dependent selection of Wilson disease models according to experimental questions.

Experimental question	Preferred first-line model	Orthogonal/confirmatory model	Models with limited suitability	Rationale
Early copper dysregulation before overt injury (38, 42, 44, 52, 54)	tx-j mouse; early Atp7b−/− mouse	ATP7B-KO HepG2/C3a under low copper; WD iPSC-HLC	LEC rat during acute hepatitis; end-stage tx mouse	Early models capture copper imbalance, metabolic remodeling, proteostasis stress, and epigenetic changes before extensive hepatocyte death
Physiological ATP7B trafficking and copper export (54, 59)	WT HepG2/C3a copper-overload model	Corrected/polarized iPSC-HLC when available	HSC-only models; LX-2	WT hepatocyte-like models define copper-induced ATP7B relocalization, lysosomal exocytosis, and adaptive copper export
Protein misfolding and ER/proteostasis stress (52–54)	ATP7B-H1069Q cells; ATP7B-KO HepG2/C3a under low copper	H1069Q iPSC-HLC or isogenic corrected line	Acute LEC rat decompensation stage	These systems allow analysis of mutation-specific folding defects, ER retention, ERAD, and early copper-sensitive proteome stress
Mitochondrial copper toxicity (29, 40, 61)	LEC rat; ATP7B-deficient rat system	ATP7B-KO hepatocytes under copper stress	HSC-only copper models	Rat models show prominent mitochondrial copper accumulation, cristae disruption, membrane damage, and respiratory-chain injury
Acute liver failure-like WD (29, 39, 40)	LEC rat; ATP7B-deficient rat system	Atp7b−/− mouse only for chronic/adjacent endpoints	tx-j mouse; low-copper ATP7B-KO cell models	Severe rat models reproduce acute decompensation and enable testing of rapid decoppering strategies such as methanobactin
Chronic hepatitis and fibrosis (39, 41, 43, 45)	Atp7b−/− mouse; tx mouse	LEC survivors; Labrador retriever copper hepatopathy as comparative model	Simple hepatocyte monoculture	Chronic *in vivo* models better reproduce progressive inflammation, tissue remodeling, extracellular matrix deposition, and long-term copper-associated hepatopathy
Hepatic steatosis and metabolic remodeling (38, 42, 44, 47)	Atp7b−/− mouse; tx-j mouse	ATP7B-KO hepatocytes as mechanistic support	HSC-only models	These models capture copper-driven alterations in lipid metabolism, methionine metabolism, methylation, and hepatic metabolic rewiring
Hepatic inflammation and stress signaling (47, 48, 60)	Atp7b−/− mouse; R778L mouse	ATP7B-KO hepatocytes plus copper; primary WD hepatocytes	WT copper-overload models alone	ATP7B-deficient systems better connect copper retention to NF-κB/AP-1 activation, oxidative stress, autophagy, and inflammatory mediator production
Direct HSC activation by copper (55, 62)	CuSO_4_-treated LX-2	iPSC-derived HSC exposed to a defined copper context as an emerging humanized extension	HepG2 monoculture	These systems test whether copper can directly activate stellate-cell fibrogenic programs
Hepatocyte-driven secondary HSC activation (52, 55, 60)	ATP7B-KO hepatocytes under copper stress	Proposed conditioned-medium or co-culture extension using HSCs/iPSC-derived HSCs	HSC monoculture alone	Co-culture or conditioned-medium systems connect ATP7B-deficient hepatocyte stress to secondary HSC activation
HSC–LSEC crosstalk and sinusoidal remodeling (34, 65)	Copper-treated HSC–LSEC co-culture	Future ATP7B-deficient multicellular/liver-on-chip confirmation	Hepatocyte-only models	These models examine VEGFA signaling, PDGFRβ/ERK activation, and capillarization-like vascular remodeling
Variant-specific therapy (50, 63)	Variant-matched cellular model (H1069Q or R778L as appropriate)	Atp7b-R780L knock-in mouse for the human R778L-corresponding background; H1069Q patient-derived/corrected iPSC-HLC for H1069Q-specific correction	Generic WT copper-overload models	Variant-specific models are required to test mutation-dependent folding, trafficking, stability, transport, or rescue; they do not establish a deterministic clinical phenotype
AAV or gene-replacement benchmarking (23, 50, 51)	Atp7b−/− mouse	R780L knock-in mouse; patient-derived/corrected WD iPSC-HLC as an orthogonal cellular platform	LX-2 or HSC-only systems	*In vivo* ATP7B-deficient models allow evaluation of hepatic delivery, copper correction, durability, and safety of gene therapy
Antifibrotic screening (34, 62)	CuSO_4_-treated LX-2 or iPSC-HSC for initial screen	ATP7B-KO hepatocyte–HSC/HSC–LSEC system, followed by Atp7b−/− or tx *in vivo* validation	WT HepG2 copper overload alone	Simplified systems are useful for screening, but validation should include ATP7B-deficient and multicellular or *in vivo* models
Long-term comparative copper hepatopathy (39, 43, 45)	Labrador retriever copper-associated hepatitis; late Atp7b−/− mouse	LEC survivors	Short-term copper-treated cell lines	Naturally occurring or chronic *in vivo* models are more appropriate for modifier genetics, longitudinal biomarkers, and long-term copper-associated liver injury
Integrated human multicellular modeling (54, 55, 64–67)	ATP7B-deficient human multicellular organoid/liver-on-chip when validated	Non-WD-specific organoid/chip systems as engineering precedents; WD iPSC-HLC and iPSC-derived HSC systems as component-level validation	Single-cell-type models	Advanced human platforms can integrate polarity, perfusion, multicellular injury, inflammation, sinusoidal dysfunction, and fibrogenic remodeling, but direct WD-specific ATP7B-deficient versions remain underdeveloped

## Literature search and framework development

A structured narrative search was conducted using PubMed as the primary database. “Wilson disease” or “ATP7B” was combined with terms including liver/hepatic injury, animal model, toxic milk, LEC, Atp7b knockout/knock-in, hepatocyte, iPSC, organoid, liver-on-chip, fibrosis, hepatic stellate cell, liver sinusoidal endothelial cell, gene therapy, mRNA, genome editing, and variant-specific therapy. Searches were updated through August 2026 and supplemented by backward and forward citation tracking. Priority was given to original studies involving ATP7B deficiency, disease-associated variants, or patient-derived WD backgrounds. ATP7B-intact systems exposed to exogenous copper were considered copper-toxicity or physiological copper-trafficking reference models rather than direct WD models. Organoid, liver-on-chip, and fibrosis studies from other liver diseases were included only when they provided transferable methodological insights and were explicitly identified as non-WD-specific technology references. Neurologic-only models and studies that did not materially inform hepatic model characterization or selection were outside the principal scope.

Experimental models were evaluated across five domains: genetic fidelity, hepatic disease-stage representation, dominant mechanism or key readout, cellular and tissue complexity, and translational objective. Disease-stage assignment was not based on hepatic copper content alone but on the combined assessment of copper accumulation, histopathological changes, mitochondrial and metabolic injury, inflammatory and fibrotic features, and disease progression. Because these pathological processes overlap temporally and mechanistically, stage assignments indicate the experimental context in which each model is most informative rather than mutually exclusive disease stages or strict clinical stages of human WD.

When multiple models could address the same question, the preferred model was selected according to the genetic fidelity, biological complexity, and decisive experimental endpoint required by the hypothesis, with complementary models carrying different limitation profiles used for orthogonal validation. These principles formed the basis of the stage-, mechanism-, genetic-fidelity-, tissue-complexity-, and translational-objective-oriented model-selection framework used throughout this Review.

## A stage-, mechanism-, and objective-oriented framework

### Wilson disease hepatic injury as a stage-dependent process

Wilson disease-associated liver injury should be viewed as a dynamic and stage-dependent pathological continuum rather than a single disease state. Although ATP7B dysfunction and hepatic copper retention represent the initiating events, the biological processes driving liver injury evolve considerably during disease progression. Clinical manifestations range from asymptomatic copper accumulation and mild steatosis to chronic hepatitis, fibrosis, cirrhosis, acute liver failure-like episodes, and, in some cases, hepatocellular carcinoma (9, 13, 35). These diverse phenotypes arise from sequential and partially overlapping processes involving copper dysregulation, hepatocellular stress responses, mitochondrial injury, metabolic remodeling, inflammation, activation of non-parenchymal cells, and tissue remodeling (7, 25, 26, 28).

Recognizing this temporal and mechanistic heterogeneity is essential for interpreting experimental findings. Different models do not merely represent varying degrees of disease severity but rather capture distinct biological stages of WD-associated hepatic injury. Consequently, experimental results obtained from one model cannot automatically be generalized across the entire disease spectrum. A stage-oriented framework of WD liver disease progression and its implications for experimental modeling are summarized in **Figures 1** and **2**.

**Figure 2:**
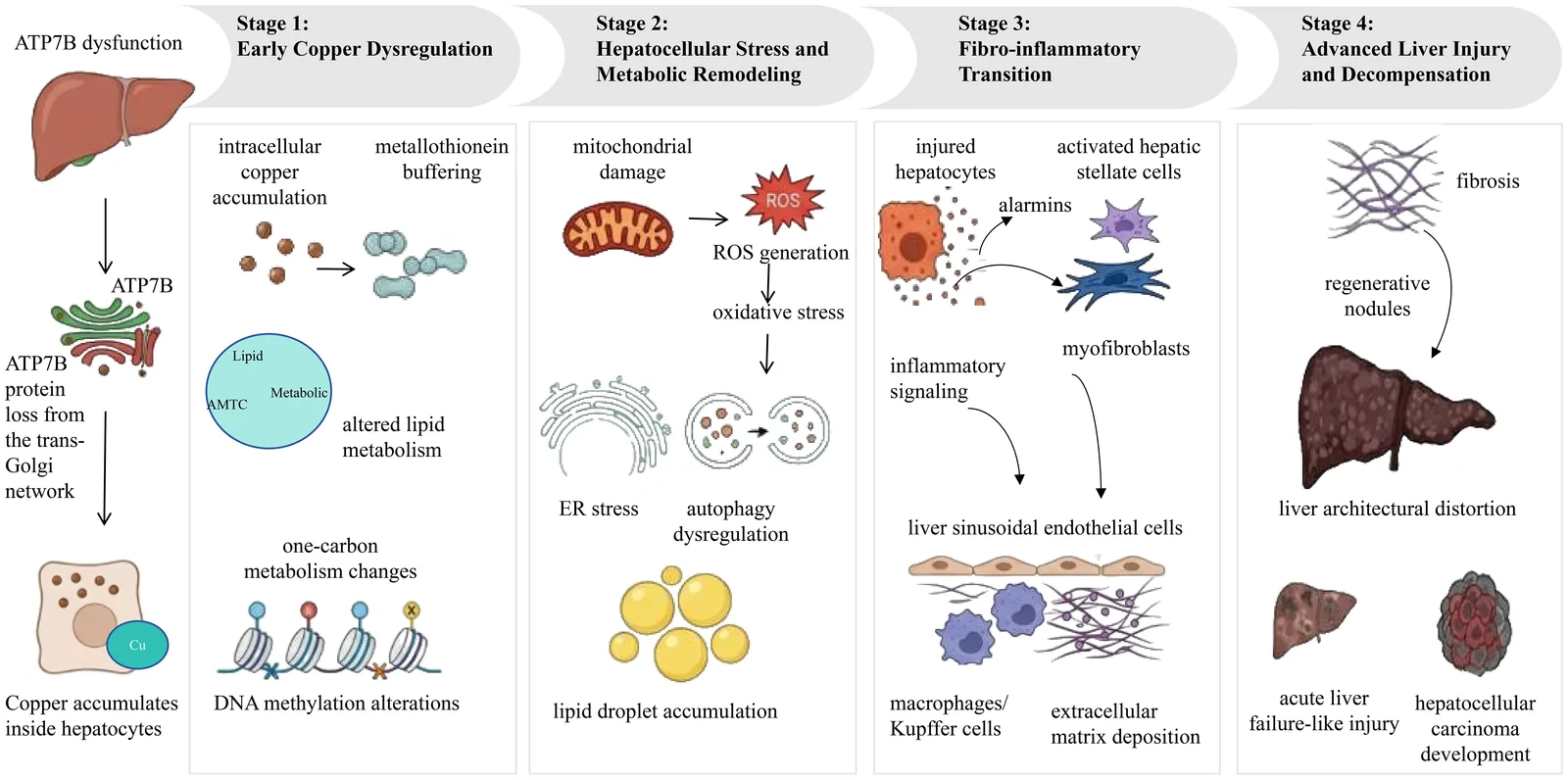
Stage-dependent progression of hepatic injury in Wilson disease and corresponding biological mechanisms. ATP7B dysfunction causes progressive hepatic copper accumulation and injury across four overlapping stages. Stage 1, early copper dysregulation: copper accumulation is accompanied by metallothionein buffering and metabolic and epigenetic alterations. Stage 2, hepatocellular stress and metabolic remodeling: mitochondrial dysfunction and ROS generation promote oxidative/ER stress, altered autophagy, and lipid-droplet accumulation. Stage 3, fibro-inflammatory transition: hepatocyte injury signals activate hepatic stellate cells, macrophages/Kupffer cells, and liver sinusoidal endothelial cells, promoting inflammation and extracellular matrix deposition. Stage 4, advanced liver injury and decompensation: fibrosis, regenerative nodules, and architectural distortion may progress to acute liver failure-like injury or hepatocellular carcinoma. These stages represent an overlapping disease continuum rather than discrete pathological states.

## Criteria for stage assignment and model preference

The stage assignment in this framework is based on the phenotype that is most reproducible and experimentally informative in each model, not on an assumption that any model reproduces a discrete human stage in full. Early-stage assignment requires copper retention with limited overt tissue destruction and measurable adaptive, metabolic, proteostatic, or epigenetic responses. The hepatocellular-stress stage is anchored by mitochondrial, redox, autophagic, inflammatory, or cell-injury readouts. Fibro-inflammatory assignment requires evidence of non-parenchymal activation and/or extracellular-matrix remodeling. Advanced-stage assignment requires severe architectural injury, decompensation, cirrhosis-like change, or an acute liver failure-like phenotype.

When two models show overlapping phenotypes, model preference is determined by the primary requirement of the research question. ATP7B genetic relevance is prioritized for WD-specific pathogenesis studies; temporal controllability for investigating early adaptive mechanisms; multicellular composition for fibrosis-related processes; severe and reproducible injury for evaluating emergency decoppering strategies; and human variant context with functional rescue capacity for mutation-specific therapeutic development. This rationale forms the basis of the model-selection workflow and comparative framework presented in the subsequent sections.

## ATP7B dysfunction and hepatic copper retention

ATP7B is the central regulator of hepatic copper homeostasis. Under physiological conditions, ATP7B delivers copper to the trans-Golgi network for ceruloplasmin biosynthesis and facilitates biliary copper excretion when intracellular copper concentrations increase (4, 5). Through these complementary functions, hepatocytes maintain copper balance despite fluctuations in dietary intake and metabolic demand.

Loss of ATP7B function impairs both ceruloplasmin maturation and biliary copper elimination, resulting in progressive hepatic copper retention. Initially, excess copper is buffered by metallothioneins, glutathione, and other copper-binding molecules. However, as intracellular copper accumulates beyond buffering capacity, toxic copper species increasingly interfere with protein folding, mitochondrial respiration, redox homeostasis, lipid metabolism, and intracellular signaling pathways (7, 11). Consequently, hepatocytes become the primary site of disease initiation and the major source of downstream pathological signals that drive inflammation and fibrosis.

This pathogenic sequence has important implications for experimental design. Models carrying ATP7B mutations, deletions, knock-in variants, or patient-derived ATP7B-deficient backgrounds directly reproduce the genetic basis of WD and are therefore most appropriate for investigating disease-specific mechanisms. By contrast, exogenous copper-overload systems primarily reflect cellular responses to copper excess and should be interpreted as copper-toxicity reference models rather than direct representations of ATP7B-deficient WD pathogenesis (17, 24, 36).

## A stage-based framework of wd hepatic injury

Based on clinical observations and experimental evidence, WD-associated liver injury can be conceptualized as four interconnected stages: (i) early copper dysregulation, (ii) hepatocellular stress and metabolic remodeling, (iii) fibro-inflammatory transition, and (iv) advanced liver injury and decompensation. Although these stages frequently overlap and progression rates vary among individuals, this framework provides a useful structure for linking biological mechanisms to experimental models and translational applications (6, 13–17).

## Early copper dysregulation

The earliest phase of WD is characterized by impaired copper homeostasis before the development of overt liver injury. At this stage, hepatocytes remain largely viable and adaptive responses predominate. Cellular changes include alterations in copper-binding proteins, proteostasis regulation, methionine metabolism, lipid metabolism, mitochondrial function, and epigenetic regulation (10, 35, 37, 38).

Importantly, the key biological question during this phase is not how copper induces cell death, but rather how hepatocytes initially adapt to chronic copper retention and what molecular events determine the transition from compensation to injury. These early alterations may establish the foundation for later inflammatory and fibrotic responses.

## Hepatocellular stress and metabolic remodeling

As hepatic copper burden increases, adaptive mechanisms become insufficient and hepatocellular stress responses intensify. This stage is characterized by mitochondrial dysfunction, oxidative stress, impaired energy metabolism, autophagy activation, lipid accumulation, inflammatory signaling, and progressive hepatocyte injury (7, 9).

Among intracellular targets, mitochondria appear particularly vulnerable. Copper accumulation disrupts mitochondrial architecture and respiratory-chain function, resulting in ATP depletion, increased reactive oxygen species generation, and amplification of cellular stress pathways (9–11). Consequently, hepatocellular injury becomes increasingly self-reinforcing and contributes to the release of inflammatory and profibrotic mediators.

## Fibro-inflammatory transition

A defining feature of disease progression is the transition from predominantly hepatocyte-centered injury to multicellular tissue remodeling. Persistent hepatocellular stress promotes activation of hepatic stellate cells, dysfunction of liver sinusoidal endothelial cells, recruitment of inflammatory cells, and progressive extracellular matrix deposition (19, 25, 34).

At this stage, fibrosis emerges not as a direct consequence of copper accumulation alone but as the result of coordinated interactions among multiple hepatic cell populations. Therefore, understanding WD-associated fibrogenesis requires consideration of hepatocyte–non-parenchymal cell communication, inflammatory signaling networks, and alterations in the hepatic microenvironment.

## Advanced injury, decompensation, and tumorigenesis

The final stage encompasses advanced fibrosis, cirrhosis, severe hepatic dysfunction, acute liver failure-like presentations, hepatocellular carcinoma may occur in a subset of patients but should not be considered an inevitable endpoint of this sequence (6, 13). At this point, pathological processes extend beyond cellular stress and involve widespread architectural disruption of liver tissue. Acute decompensation may occur through sudden amplification of mitochondrial injury, oxidative stress, inflammatory cascades, and systemic metabolic imbalance (11). In parallel, long-standing copper retention and chronic inflammation can promote progressive fibrogenesis and may contribute to tumorigenic processes in selected contexts. These advanced manifestations reflect, but do not necessarily represent a fixed linear endpoint of, pathological processes initiated much earlier during disease progression.

## Implications for model selection

The stage-dependent nature of WD has important consequences for experimental research. Mechanisms identified in advanced disease models may not accurately reflect early pathogenic events, whereas findings derived from simplified copper-exposure systems may not represent ATP7B-deficiency-driven pathology. Consequently, model selection should be guided by the specific biological process under investigation rather than by assumptions regarding overall disease severity.

Within this framework, early-stage models are most appropriate for studying copper adaptation, metabolic remodeling, and proteostasis stress; intermediate-stage models facilitate investigation of mitochondrial dysfunction and inflammatory signaling; multicellular platforms are particularly relevant for fibro-inflammatory transition; and advanced *in vivo* systems are required to evaluate decompensation, cirrhosis, and therapeutic efficacy in severe disease. This stage-oriented perspective provides the conceptual foundation for the model-specific discussions presented in the following sections.

## Practical model-selection algorithm

Model selection can therefore proceed through four sequential filters. The first filter defines whether the question is WD-specific pathogenesis or general copper toxicity/trafficking; the former requires an ATP7B-deficient or disease-variant background. The second identifies the target stage or dominant mechanism. The third identifies the minimum biological complexity needed to measure the decisive endpoint—for example, hepatocyte-autonomous signaling, hepatocyte–HSC communication, sinusoidal remodeling, or whole-organism copper excretion. The fourth selects an orthogonal confirmatory model with a different limitation profile. For therapeutic studies, a final translational filter asks whether the model can measure functional target correction, durability, toxicity, and the clinically relevant disease endpoint.

## *In vivo* models: mapping animal systems to disease stages

Animal models are core tools in WD liver research because they can capture hepatic copper accumulation, biliary copper excretion defects, systemic copper metabolism, immune responses, fibrosis progression, and whole-body responses to therapeutic interventions. However, different WD animal models are most informative for different stage-associated phenotypes and mechanistic questions rather than corresponding to discrete human disease stages. Severe Atp7b-deficient rat models, including the LEC rat, are particularly informative for severe liver injury and acute decompensation, tx-j mice are better suited for early copper accumulation and metabolic remodeling, Atp7b−/− mice serve as standardized chronic models, tx mice are useful for chronic copper toxicity and progressive injury, and Labrador retriever copper-associated hepatitis provides a naturally occurring large-animal model of long-term copper-related liver disease (14, 16, 17, 29, 38–44). The major *in vivo* models, their genetic backgrounds, hepatic phenotypes, relevant research questions, and limitations are summarized in **Table 2**.

**Table 2:** Established and comparative animal models of Wilson disease-associated hepatic injury.

Model category	Representative model	Genetic basis/model status	Hepatic phenotype and disease stage	Main mechanistic and translational utility	Preferred use/when to choose	Key limitations
Spontaneous severe rat model (16, 39, 40)	LEC rat	Spontaneous Atp7b deletion; established WD-like animal model	Marked hepatic copper accumulation, low ceruloplasmin, acute or chronic hepatitis, fibrosis, and hepatocellular carcinoma in survivors	Strong model for severe hepatic injury, mitochondrial copper toxicity, acute hepatic decompensation, and inflammation–fibrosis–tumor progression	Choose when the primary endpoint is severe hepatocellular injury, mitochondrial rescue, acute decompensation, or rapid decoppering; preferred over slow mouse models for acute therapeutic windows	Represents the severe hepatic end of WD; iron dysregulation and strong tumor predisposition may complicate interpretation
Spontaneous toxic milk mouse model (17, 41)	tx mouse	Spontaneous Atp7b mutation; established toxic milk mouse model	Severe hepatic copper retention, chronic hepatitis, parenchymal injury, and progression toward cirrhosis-like pathology	Useful for studying high-burden hepatic copper toxicity, advanced liver injury, and chronic copper-associated damage	Choose for sustained high-burden copper hepatopathy and chronic injury; preferred over tx-j when a more advanced chronic phenotype is required	Phenotype is influenced by neonatal nursing conditions, strain background, and colony history
Slow-progressing toxic milk mouse model (38, 41)	tx-j mouse	Atp7b G712D mutation; established spontaneous mouse model	Progressive hepatic copper loading, steatosis, inflammation, early fibrotic changes, altered methionine metabolism, and DNA methylation changes	Suitable for early-stage WD biology, metabolic remodeling, one-carbon metabolism, epigenetic changes, and diet–copper interactions	Choose for slow early-stage adaptation, one-carbon metabolism, and epigenetic remodeling; preferred over LEC/tx models when overt severe injury would confound early mechanisms	Less suitable for fulminant hepatitis or acute liver failure-like WD
Standardized knockout mouse model (14, 42–44, 47)	Atp7b−/− mouse	Germline Atp7b knockout; established engineered WD model	Hepatic copper accumulation, steatosis, metabolic remodeling, hepatitis, fibrosis, and age-dependent chronic liver injury	Widely used for stage-resolved pathogenesis, omics profiling, therapeutic testing, and preclinical gene-therapy benchmarking	Default general-purpose *in vivo* WD model for stage-resolved studies, omics, long-term treatment, and gene-therapy benchmarking; use age-defined cohorts to match the target stage	Disease severity varies with knockout allele, background strain, sex, age, diet, microbiome, and husbandry conditions
Severe mitochondria-centered rat model (29, 40)	Atp7b-defective/Atp7b-deficient rat systems	Atp7b-deficient rat models; established for mitochondrial copper toxicity and acute hepatic injury	Hepatic copper overload with mitochondrial copper mislocalization, cristae disruption, respiratory-chain impairment, oxidative injury, and acute liver failure-like phenotype	Particularly valuable for mitochondrial injury, organelle-level copper toxicity, and rapid decoppering strategies such as methanobactin	Choose when organelle-level mitochondrial toxicity, larger-tissue sampling, pharmacokinetics, or rapid decoppering efficacy is the decisive endpoint	Less widely available than mouse models; biased toward severe acute-stage biology
Variant-specific precision mouse model (50)	Atp7b-R780L knock-in mouse	Murine Atp7b-R780L knock-in corresponding to the human ATP7B R778L variant; direct genotype-specific WD model	Murine Atp7b-R780L knock-in corresponding to the human ATP7B R778L variant; direct genotype-specific WD model	Murine Atp7b-R780L knock-in corresponding to the human ATP7B R778L variant; direct genotype-specific WD model	Choose when efficacy depends on the presence of a defined missense ATP7B protein; not preferred for inferring deterministic human clinical progression	One variant cannot represent the full ATP7B mutational spectrum; phenotype may depend on genetic background and line design
Naturally occurring comparative large-animal model (45)	Labrador retriever copper-associated hepatitis/copper toxicosis	ATP7B risk variant with ATP7A modifier; comparative copper hepatopathy rather than classical monogenic WD	Adult-onset chronic copper-associated hepatitis, hepatic copper accumulation, and fibrosis	Valuable for modifier genetics, ATP7A–ATP7B interaction, long-term copper retention, dietary copper effects, and comparative hepatology	Choose for long-term comparative copper hepatopathy, dietary exposure, and modifier genetics in a large-animal context; not as a strict monogenic WD substitute	Not a strict monogenic WD model; neurological WD manifestations are not typical; breed-specific modifiers limit direct extrapolation

## Severe hepatic injury and acute decompensation models

Severe hepatic injury and acute decompensation models are primarily used to investigate the most advanced manifestations of WD liver disease, including mitochondrial copper toxicity, acute liver failure-like presentations, extensive hepatocellular injury, and rapid decoppering strategies. Although these models are not optimal for studying early copper dysregulation, they are highly valuable for understanding organelle pathology and therapeutic windows in severe WD (16, 29, 39, 40).

## LEC rat

The Long-Evans Cinnamon (LEC) rat is a classical spontaneous Atp7b-deficient animal model characterized by marked hepatic copper accumulation, reduced serum ceruloplasmin, acute or chronic hepatitis, fibrosis, and a predisposition to hepatocellular carcinoma in surviving animals (16, 39). One defining feature of the LEC rat is the severity of liver injury, with animals developing acute hepatitis and hepatic decompensation during specific stages of disease progression. Consequently, this model is widely used to represent the severe end of the WD hepatic disease spectrum.

Mechanistically, the LEC rat is particularly useful for studying mitochondrial copper toxicity. Hepatic copper overload results in abnormal mitochondrial copper distribution, disruption of cristae architecture, impairment of respiratory chain function, and enhanced oxidative stress. These pathological alterations provide important mechanistic insights into acute liver injury and rapid deterioration of hepatic function. In addition, surviving animals may progress to chronic hepatitis, fibrosis, and hepatocellular carcinoma, making the model suitable for studying the links among chronic copper toxicity, inflammation, fibrosis, and tumorigenesis (16, 29, 39, 40).

From a translational perspective, the LEC rat has been extensively used to evaluate rapid decoppering therapies and mitochondrial protective interventions. Methanobactin, a high-affinity copper-binding compound, reversed acute liver failure-like injury in a rat model of WD, supporting the value of severe rat models for testing emergency copper-removal strategies (29). However, the LEC rat also has notable limitations. Its severe phenotype, strong tumor susceptibility, and potential abnormalities in iron metabolism may reduce its ability to represent the natural disease course of most WD patients. Therefore, the LEC rat should be considered primarily a model of severe liver injury and acute decompensation rather than a general model of early WD pathology (16, 29, 39, 40).

## Other Atp7b-deficient rat systems

In addition to the LEC rat, other experimentally characterized Atp7b-deficient rat systems, where specifically defined by strain or allele, may provide complementary platforms for studying severe WD-like liver injury.These models typically exhibit profound hepatic copper overload, mitochondrial copper mislocalization, structural mitochondrial abnormalities, respiratory chain dysfunction, oxidative damage, and acute liver failure-like phenotypes (29, 40). Compared with mouse models, rats offer practical advantages because their larger liver size facilitates studies of biliary copper excretion, mitochondrial function, pharmacokinetics, and tissue copper distribution.

Severe Atp7b-deficient rat models have been particularly valuable for evaluating rapid decoppering strategies such as methanobactin. As a high-affinity copper-binding molecule, methanobactin can rapidly mobilize excess tissue copper and improve mitochondrial injury and hepatic function in the setting of severe copper overload (29). Consequently, Atp7b-deficient rat systems provide an important platform for validating therapeutic approaches that target acute copper toxicity. Their major strengths include severe pathology, clearly defined therapeutic windows, and robust efficacy readouts. However, because they predominantly represent acute and advanced disease stages, they are less suitable for modeling the slow progression of early WD liver injury (16, 29).

## Chronic copper hepatopathy and fibrosis models

Chronic copper hepatopathy models are particularly useful for studying persistent copper retention, chronic inflammation, fibrosis, cirrhosis-like changes, dietary copper effects, and long-term therapeutic interventions. Their translational value lies in their ability to mimic the gradual progression experienced by many WD patients, from asymptomatic copper accumulation to chronic liver disease (14, 17, 38, 41–49).

## tx mouse

The tx mouse is a spontaneous Atp7b mutant model characterized by substantial hepatic copper accumulation, chronic hepatocellular injury, inflammation, and progressive liver disease (17, 41). Compared with the tx-j mouse, the tx mouse generally develops more severe copper retention and hepatic injury, making it more suitable for studying chronic copper toxicity and progressive liver damage than for investigating early metabolic alterations. The primary value of the tx mouse lies in its ability to model sustained copper burden and chronic liver pathology. It provides an effective system for examining how long-term copper accumulation perpetuates inflammation, promotes tissue injury, and drives fibrogenesis. The model is also useful for evaluating conventional decoppering therapies, dietary copper interventions (43, 44, 47), and disease-modifying treatments. Nevertheless, phenotypic variability may be influenced by genetic background, housing conditions, and breeding history. Therefore, studies using tx mice should carefully report strain background, age, sex, dietary copper content, and pathological endpoints (17, 41).

## Atp7b^−/−^ mouse

The Atp7b^−/−^ mouse is currently one of the most widely used genetically engineered models of WD. Disruption of the Atp7b gene causes impaired biliary copper excretion, resulting in hepatic copper accumulation, steatosis, hepatocellular injury, inflammation, metabolic remodeling, and age-dependent fibrosis. Because of its defined genetic manipulation, experimental tractability, and compatibility with standardized longitudinal studies, the Atp7b−/− mouse has become a central model for mechanistic studies and therapeutic evaluation.

One major advantage of the Atp7b−/− mouse is its ability to capture several stage-associated features of WD liver injury. Early-stage animals can be used to investigate copper accumulation, steatosis, and metabolic remodeling, whereas later-stage animals are suitable for studying chronic inflammation, fibrosis, and treatment responses (14, 42–44, 47). Recent work further refined the histological timeline of liver disease development in Atp7b^−/−^ mice, supporting their use for stage-resolved studies of WD-associated hepatic injury (43). The model is particularly amenable to transcriptomic, proteomic, metabolomic, epigenomic, and metallomic approaches, enabling temporal maps of disease progression (42, 44, 47).

In translational studies, Atp7b^−/−^ mice are frequently used to evaluate conventional decoppering therapy, zinc treatment, AAV-mediated gene therapy, mini-ATP7B constructs, mRNA-based therapies, and genome-editing approaches (14, 42–44, 47, 50, 51). However, important limitations should be recognized. Complete gene knockout is not fully equivalent to the many disease-causing missense variants observed in WD patients. Furthermore, phenotype severity may be influenced by knockout allele design, genetic background, sex, age, dietary copper intake, gut microbiota composition (44, 47), and environmental conditions. Therefore, the Atp7b−/− mouse should be viewed as a widely used general WD model suitable for standardized study designs, rather than a representation of all ATP7B variants and clinical phenotypes.

## LEC survivors

Surviving LEC rats that progress beyond the acute disease phase may develop chronic liver disease, fibrosis, cirrhosis-like changes, and hepatocellular carcinoma (16, 29, 39, 40). These animals provide a unique opportunity to investigate the interactions among chronic copper toxicity, tissue repair, ECM deposition, persistent inflammation, and carcinogenesis. Unlike animals experiencing acute hepatitis, LEC survivors allow researchers to examine how long-term copper retention contributes to fibrogenesis and tumor development.

Interpretation of findings from LEC survivors should nonetheless be cautious. Because LEC rats possess a strong inherent tendency toward hepatocellular carcinoma and may harbor additional non-copper-related oncogenic influences, they are most appropriately used to study mechanisms by which chronic copper-associated injury promotes carcinogenesis rather than to estimate cancer risk in human WD patients (16, 39).

## Labrador retriever copper-associated hepatitis

Labrador retriever copper-associated hepatitis is a naturally occurring canine copper-related liver disease. Although it is not a classical monogenic WD model, it is associated with ATP7B risk variants and ATP7A modifier alleles and is characterized by adult-onset hepatic copper accumulation, chronic hepatitis, and fibrosis (45). Because it develops spontaneously, progresses slowly, and occurs in a large-animal setting that more closely resembles clinical disease management, this model offers unique value for comparative medicine and translational research.

Canine models are particularly useful for investigating long-term dietary copper exposure, modifier genes, ATP7A–ATP7B interactions, and the natural progression of chronic copper hepatopathy. However, important differences from human WD exist. The genetic mechanisms are not identical, neurological manifestations are generally absent or atypical, and breed-specific factors limit direct extrapolation. Accordingly, this model should be regarded as a comparative copper hepatopathy model rather than a strict WD model (45).

## Early-stage and metabolic remodeling models

Early-stage models are valuable because they capture pathological events that occur before overt hepatocyte death and extensive fibrosis. Key questions include how copper accumulation alters lipid metabolism, one-carbon metabolism, and DNA methylation; how hepatocytes maintain compensation under copper stress; and which molecular thresholds determine the transition from adaptive responses to irreversible injury (38, 42, 44, 47, 52).

## tx-j mouse

The tx-j mouse carries the Atp7b G712D mutation and typically exhibits slow-progressing hepatic copper accumulation and WD-like liver pathology (17, 39, 41). Unlike severe rat models, the tx-j mouse is particularly suited for studying early copper dysregulation, steatosis, mild inflammation, metabolic remodeling, and epigenetic alterations. This model has been especially valuable in studies of one-carbon metabolism and DNA methylation. Copper accumulation can disrupt methionine metabolism, S-adenosylmethionine synthesis, methyl donor balance, and DNA methylation patterns, thereby altering hepatocellular transcriptional programs. Because disease progression is relatively slow, investigators can observe these early metabolic and epigenetic events before the onset of severe liver injury. Consequently, the tx-j mouse is well suited for evaluating nutritional interventions, methyl donor supplementation, and dietary copper regulation (38).

## Early Atp7b−/− mouse

Early-stage Atp7b−/− mice also represent an important platform for studying the initial phases of WD. Although the same model later develops chronic inflammation and fibrosis, early time points primarily reflect hepatic copper accumulation, steatosis, subclinical mitochondrial dysfunction, and metabolic reprogramming (14). Longitudinal studies using early Atp7b−/− mice can help identify molecular transition points that mark the shift from asymptomatic copper accumulation to inflammation and fibrogenesis (42). Compared with tx-j mice, Atp7b−/− mice are more suitable for standardized experimental studies and therapeutic validation, whereas tx-j mice are particularly valuable for investigating early metabolic and epigenetic alterations in a slow-progressing mutant background (14, 38, 42–44, 47). Together, these models provide complementary insights into the earliest stages of WD liver injury.

## Variant-specific *in vivo* models

As precision medicine advances in WD, conventional knockout models alone are no longer sufficient. Many disease-causing ATP7B alleles are missense variants rather than complete gene deletions. Variant-specific animal models are therefore particularly important for defining mutation-level molecular defects, residual ATP7B function, and treatment responsiveness; their value for clinical genotype–phenotype inference is more limited because human disease expression is strongly modified by allelic combinations and non-genetic factors (49, 53, 54).

## Atp7b-R780L knock-in mouse

The Atp7b-R780L knock-in mouse carries the murine R780L substitution corresponding to the human ATP7B R778L variant, a clinically relevant missense variant prevalent in East Asian populations (50). Unlike Atp7b−/− mice, knock-in models retain expression of mutant ATP7B protein, which may make them particularly informative for investigating residual function and mutation-specific therapeutic responses; folding, localization, and stability defects should be attributed to the model only when directly demonstrated.

Major applications include variant-function analyses, evaluation of mutation-compatible gene therapies, and testing of rescue strategies that require residual mutant ATP7B protein. For some missense mutations, pharmacological chaperones, proteostasis regulators, or trafficking rescue approaches may partially restore ATP7B function, whereas knockout models cannot adequately evaluate such interventions (49, 53, 54). However, single-variant models have inherent limitations. The ATP7B mutational spectrum is highly diverse, and different variants may cause distinct defects involving protein folding, trafficking, catalytic activity, or protein degradation. Consequently, the R780L model represents an *in vivo* model corresponding to the human R778L variant but cannot represent H1069Q or other ATP7B variants with distinct molecular defects (17, 49, 53, 55). Future efforts should focus on developing additional knock-in models representing common clinical variants and integrating them with patient-derived iPSC-HLCs and isogenic correction systems to establish a more comprehensive genotype–phenotype map (14, 55). Accordingly, variant-specific experimental phenotypes should be separated from claims about clinical disease progression.

## Interpreting variant-specific models: molecular phenotype versus clinical phenotype

The evidence supports a distinction between experimentally robust variant-level molecular phenotypes and less stable clinical genotype–phenotype associations. H1069Q systems reproducibly model misfolding, ER retention, impaired trafficking, and degradation-prone ATP7B behavior. R778L cellular systems have revealed copper-stress responses involving autophagy and necroptosis, whereas the R780L knock-in mouse provides an *in vivo* missense background for therapeutic testing. In clinical cohorts, however, the same ATP7B variant can occur in different allelic combinations and phenotypic contexts. A large European cohort found no consistent relationship between individual ATP7B mutations and hepatic versus neurologic presentation or cirrhosis, while a 2023 meta-analysis in China associated R778L with earlier presentation and lower ceruloplasmin but not with the type of first clinical presentation. A 2025 registry study further demonstrated variable penetrance among frequent ATP7B variants (56–58).

Accordingly, this Review uses “variant-specific” to denote a defined molecular model and avoids assigning a fixed clinical progression trajectory to H1069Q, R778L, R780L, or other single variants. Variant-informed precision therapy should be based on the functional defect demonstrated in the relevant model and, where possible, confirmed in patient-derived or isogenic systems. **Table 3** summarizes the experimentally supported molecular phenotype, best-suited application, and clinical interpretation boundary for H1069Q, R778L, and R780L model systems.

**Table 3:** Variant-specific experimental models and boundaries of clinical interpretation.

ATP7B variant/model	Experimentally supported molecular phenotype	Best-suited application	Clinical interpretation boundary
H1069Q cellular and iPSC-HLC systems (54)	Misfolding, ER retention, impaired copper-responsive trafficking, instability/degradation; CRISPR/Cas9 gene correction in patient-specific iPSC-derived HLCs restores ATP7B trafficking and increases copper resistance	Chaperone/proteostasis rescue, trafficking rescue, isogenic gene correction, mutation-specific functional assays	Historical studies reported later/neurologic presentation in some cohorts, but large multicenter data do not support deterministic prediction of presentation or cirrhosis from individual ATP7B mutations
R778L hepatocyte systems (49)	Copper-stress adaptation involving autophagy and altered necroptosis responses	Mechanistic analysis of copper tolerance/stress pathways and testing of R778L-compatible rescue strategies	A 2023 Chinese meta-analysis associated R778L with earlier presentation and lower ceruloplasmin, but not with hepatic versus neurologic first presentation; clinical inference should therefore remain population- and context-specific
Atp7b-R778L mouse model (48)	Abnormal copper metabolism, hepatic inflammation and fibrosis, together with neurological manifestations in the reported model	*In vivo* analysis of R778L-associated copper dysregulation, hepatic inflammation, liver–brain interactions, and disease mechanisms	Use according to the nomenclature and phenotype reported by Dong et al.; do not assume equivalence with all R778L/R780L model designs or infer a deterministic human phenotype
Atp7b-R780L knock-in mouse (50)	Murine R780L substitution corresponding to human ATP7B R778L; abnormal copper metabolism with a relatively mild hepatic phenotype in the reported KI model	Variant-compatible *in vivo* therapy, AAV8-ΔC4ATP7B benchmarking, and copper-handling endpoints in a defined missense background	The murine R780L KI is an experimental model corresponding to human ATP7B R778L; its phenotype should not be generalized to all ATP7B variants or interpreted as a fixed human disease trajectory

## Hepatocyte-based models: dissecting ATP7B-dependent cellular mechanisms

Because hepatocytes are the initiating cell type in WD liver injury, hepatocyte-based models are critical for investigating ATP7B-dependent cell-autonomous mechanisms. Compared with animal models, hepatocyte systems provide greater control over copper exposure, treatment duration, genetic background, and experimental manipulation, making them ideal for mechanistic studies, drug screening, and mutation-specific investigations (49, 52–54, 59, 60). However, substantial differences exist among hepatocyte models in terms of WD specificity. Wild-type copper-overload systems, ATP7B-knockout cells, mutation-specific models, patient-derived iPSC-HLCs, and primary WD hepatocytes should not be considered equivalent. The major hepatocyte-based models and their applications are summarized in **Table 4**.

**Table 4:** Hepatocellular models of ATP7B dysfunction and copper-induced injury.

Model type	Representative model	WD specificity	Core phenotype	Main utility	Interpretive boundary/preferred use	Key limitations
Copper-overload reference model (59)	WT HepG2/C3a copper overload	Copper-toxicity/physiological trafficking reference model	Copper-induced ATP7B relocalization from the Golgi toward vesicular, lysosomal, or canalicular compartments; lysosomal exocytosis contributes to copper export	Defines physiological ATP7B trafficking and adaptive copper-export responses in hepatocyte-like cells	Use as a copper-toxicity/trafficking comparator; do not infer WD-specific consequences unless contrasted with an ATP7B-deficient background	Not ATP7B-deficient; tumor-derived cells lack full hepatocyte polarity and mature bile canalicular architecture
ATP7B-deficient hepatocyte model (60, 61)	HepG2 ATP7B-KO plus copper	Direct ATP7B-deficient cellular model	Copper-induced oxidative stress, autophagy, inflammatory signaling, and hepatocyte injury	Useful for dissecting downstream injury pathways after ATP7B loss and for drug screening	Use for ATP7B-loss mechanisms and pharmacologic screening; confirm key findings in a humanized or *in vivo* WD model	Hepatoma-derived metabolism and high copper exposure may overrepresent late toxicity
Low-copper proteostasis model (52)	HepG2/C3a ATP7B-KO under low copper stress	Direct ATP7B-deficient early-injury model	Protein misfolding and proteostasis stress at relatively low copper concentrations before overt cytotoxicity	Useful for early-stage WD mechanisms, copper-sensitive folding thresholds, and proteostasis-targeted interventions	Use for early proteostasis thresholds before overt toxicity; not a fibrosis or whole-liver model	Lacks multicellular hepatic microenvironment and fibrosis-related non-parenchymal cells
Mutation-specific folding model (53)	ATP7B-H1069Q transfected or engineered cells	Mutation-specific direct WD model	ER retention, impaired ATP7B trafficking, reduced copper-responsive relocalization, and degradation-prone ATP7B behavior	Useful for studying H1069Q folding defects and testing chaperone or trafficking-rescue strategies	Use for H1069Q folding/trafficking rescue; clinical phenotype cannot be inferred from the cellular defect alone	Overexpression or non-endogenous expression may affect localization and degradation kinetics
Mutation-specific adaptive injury model (49)	ATP7B-R778L mutant hepatocyte model	Mutation-specific direct WD model	Autophagy activation and altered necroptosis response under copper stress	Useful for studying R778L-associated cellular adaptation and mutation-specific therapeutic response	Use for R778L cellular stress and rescue phenotypes; clinical associations are population-dependent	Stable clonal models may select copper-tolerant phenotypes; findings may not generalize to all ATP7B variants
Patient-derived iPSC hepatocyte platform (54)	WD iPSC-derived hepatocyte-like cells	Patient-specific direct WD model	Impaired copper handling in patient genetic backgrounds; allows hepatic and neural differentiation platforms	Useful for personalized disease modeling, variant-specific testing, and genotype-dependent response studies	Use for patient-context response heterogeneity; pair with isogenic correction when attributing causality to a variant	Hepatocyte-like cells are often immature; differentiation efficiency and batch variability remain major limitations
H1069Q patient-specific iPSC model (54, 63)	H1069Q iPSC-derived hepatocyte-like cells	Patient-specific mutation model	Mutation-dependent ATP7B dysfunction and impaired copper handling; CRISPR/Cas9 correction restores copper resistance and ATP7B trafficking	Useful for patient-specific functional analysis, rescue studies, and gene correction	Use for H1069Q patient-specific rescue and gene correction; maturation remains an important constraint	Well-characterized homozygous or compound-heterozygous patient lines are limited; maturation remains incomplete
Gene-corrected patient-specific isogenic model (63)	H1069Q WD iPSC-HLC with CRISPR/Cas9 correction	Direct causal validation model	Restoration of copper resistance and ATP7B trafficking compared with the uncorrected patient-specific background	Useful for validating ATP7B variant causality and benchmarking mutation-specific gene correction	Preferred causal benchmark for H1069Q correction because corrected and uncorrected cells derive from the same patient-specific background	Technically demanding; clonal variability and off-target editing require rigorous controls
Primary human WD hepatocyte model (61)	WD primary hepatocytes or patient-derived liver tissue hepatocytes	Direct human disease validation model	Autophagy activation, oxidative stress, inflammatory signaling, and clinically relevant copper injury when viable cells are available	Highest clinical relevance for validating mechanisms observed in animals and cell lines	Use as high-value human validation, not as a scalable discovery platform	Scarce availability, donor variability, short culture lifespan, and confounding by advanced liver disease

## Copper-overload reference models versus ATP7B-deficient models

One of the most important distinctions in hepatocyte research is that wild-type HepG2/C3a copper-overload systems are copper-toxicity reference models, whereas ATP7B-deficient hepatocytes represent direct WD cellular models. Wild-type HepG2/C3a cells exposed to CuSO4 or other copper sources exhibit increased intracellular copper, oxidative stress, ATP7B relocalization, and adaptive copper export responses. These systems are useful for studying how normal hepatocytes respond to elevated copper and can serve as reference models for copper toxicity and physiological ATP7B trafficking. However, because ATP7B function remains intact, these models do not reproduce the fundamental pathogenic mechanism of WD, namely impaired biliary copper excretion caused by ATP7B deficiency.

By contrast, ATP7B-KO HepG2 or ATP7B-KO C3a cells more closely resemble WD at the genetic level. These models are suitable for investigating how ATP7B loss induces oxidative stress, autophagy, inflammatory signaling, mitochondrial dysfunction, and cell death. Recent ATP7B-KO cell culture work further supports their use for modeling copper-induced oxidative stress and inflammatory processes in a genetically relevant WD background. Therefore, studies focused on WD-specific hepatocyte injury should preferentially employ ATP7B-deficient systems rather than wild-type copper-overload models. Copper-induced ROS production in wild-type HepG2 cells demonstrates general copper toxicity, whereas similar findings in ATP7B-KO cells provide stronger evidence that copper accumulation directly contributes to hepatocellular injury in the context of ATP7B deficiency (49, 52, 53, 59, 60).

## ATP7B trafficking and adaptive copper export

Although wild-type HepG2/C3a copper-overload systems are not strict WD models, they remain valuable for studying physiological ATP7B trafficking. Under basal conditions, ATP7B is primarily localized to the trans-Golgi network. As intracellular copper levels increase, ATP7B traffics from the Golgi to vesicular, lysosomal, or canalicular-like compartments, thereby facilitating copper export. This trafficking process represents an adaptive response that allows hepatocytes to maintain copper homeostasis under conditions of increased copper load. Emerging evidence suggests that lysosome-related structures and lysosomal exocytosis may contribute to copper elimination.

Wild-type HepG2/C3a copper-overload models allow investigators to monitor ATP7B localization under varying copper concentrations and exposure durations, providing mechanistic insights into copper transport, vesicular trafficking, and lysosome-mediated export. However, because HepG2/C3a cells originate from hepatocellular carcinoma, they do not fully recapitulate hepatocyte polarity, canalicular organization, or mature metabolic function. Therefore, they are best viewed as mechanistic trafficking models rather than standalone WD liver injury models (59).

## Early proteostasis stress before overt toxicity

ATP7B-KO HepG2/C3a cells exposed to low copper stress conditions provide a useful model for investigating early proteostasis disturbances that occur before overt cytotoxicity. Under these conditions, copper levels are insufficient to induce widespread cell death but can trigger protein misfolding, proteostasis imbalance, mild endoplasmic reticulum stress, autophagy modulation, and adaptive stress responses (52).

The significance of this model lies in its ability to define a potential early intervention window in WD. Long before ALT elevation, overt inflammation, or extensive cell death become apparent, ATP7B-deficient hepatocytes may already exhibit detectable proteostasis abnormalities. Interventions targeting protein homeostasis, oxidative stress, metabolism, or intracellular copper buffering during this stage may theoretically prevent progression from early copper accumulation to irreversible liver injury.Nonetheless, the limitations of transformed hepatoma-derived cells, the absence of mature biliary structures, and the lack of non-parenchymal and immune cells should be recognized. Findings obtained in this model should therefore be validated in iPSC-HLCs, primary hepatocytes, or *in vivo* systems (52, 54, 60, 61).

## Mutation-specific cellular models

Mutation-specific cellular models are particularly important for investigating the pathological mechanisms of ATP7B missense variants and for developing precision therapies. Different ATP7B variants can produce distinct defects involving protein folding, ER retention, intracellular trafficking, catalytic activity, protein instability, and degradation (49, 53, 54).

## H1069Q models

H1069Q is one of the most common ATP7B variants among European and North American WD patients. Cellular models expressing H1069Q typically exhibit ATP7B protein misfolding, ER retention, impaired copper-induced trafficking, and enhanced degradation. These systems are therefore well suited for studying pharmacological chaperones, proteostasis modulators, inhibition of ER-associated degradation, and trafficking rescue strategies. Importantly, H1069Q models do not simply mimic ATP7B absence but instead model the presence of a dysfunctional mutant protein. This distinction is highly relevant for developing mutation-specific rescue therapies.

## R778L and R780L-related systems

R778L is among the most prevalent ATP7B variants in East Asian populations. Hepatocyte models carrying R778L are useful for studying copper stress-induced autophagy, necroptosis, and mutation-specific adaptive responses (49). R780L-related systems complement R780L knock-in mice by linking cellular mechanisms to *in vivo* phenotypes (48, 50). These experimental systems define molecular consequences of a given ATP7B variant but do not, by themselves, establish whether that variant predicts hepatic versus neurologic presentation or the tempo of human disease. stable cell lines may gradually select for copper-tolerant clones during long-term culture. Therefore, findings should ideally be validated using transient expression systems, gene-edited isogenic cells, and patient-derived cellular models (49, 53, 54).

## Patient-derived and gene-corrected platforms

Patient-derived iPSC-HLCs, gene-corrected isogenic systems, and primary WD hepatocytes provide powerful humanized platforms for WD modeling. WD iPSC-HLCs retain patient-specific genetic backgrounds and are well suited for investigating individualized copper-handling defects, mutation-specific ATP7B localization abnormalities, and drug responses. For H1069Q, patient-derived hepatocyte-like systems are particularly informative for protein-folding and trafficking defects and for testing whether correction or rescue restores copper-responsive ATP7B behavior (14, 59, 62).

CRISPR-mediated correction enables the generation of isogenic controls, allowing direct comparison between mutant and corrected cells within the same genetic background. Such systems provide compelling evidence for the causal role of ATP7B variants. A 2026 patient-specific H1069Q iPSC study extended this strategy by correcting the point mutation with CRISPR/Cas9 and showing recovery of copper resistance and ATP7B trafficking after hepatocyte-like differentiation (63). Gene-corrected and gene-replacement platforms also serve as valuable benchmarks for evaluating AAV vectors, mini-ATP7B constructs, mRNA therapies, and genome-editing approaches (50, 54, 61). Restoration of ATP7B expression that normalizes copper handling, improves protein localization, and reduces intracellular copper burden provides strong evidence supporting therapeutic efficacy.

Primary human WD hepatocytes possess the highest degree of clinical relevance and are important for validating findings generated in animal models and immortalized cell lines. However, their availability is extremely limited. Most samples originate from advanced liver disease or transplant tissues and are influenced by donor variability, prior treatments, disease stage, and limited survival in culture. Consequently, primary WD hepatocytes are best used for human validation of key mechanistic findings rather than large-scale screening applications (60, 61). Overall, iPSC-HLCs and gene-corrected platforms represent central components of future precision medicine strategies for WD. Nevertheless, challenges remain, including incomplete hepatocyte maturation, limited canalicular function, batch-to-batch variability, and difficulties maintaining long-term stability in culture (54).

## Modeling the fibrogenic transition in Wilson disease

Liver fibrosis in WD cannot be adequately modeled using hepatocyte monocultures or HSC monocultures alone. Rather, fibrosis represents a tissue-level process driven by interactions among ATP7B-deficient hepatocytes, activated non-parenchymal cells, inflammatory signaling networks, sinusoidal remodeling, and extracellular matrix deposition (34, 46, 55, 62, 64, 65). Therefore, future fibrosis models should ideally integrate ATP7B-deficient hepatocytes, HSCs, LSECs, immune-like cells, and ECM components. Because published WD-relevant fibrosis systems differ substantially in copper source, concentration, exposure duration, and cellular composition, reported parameters should be treated as study-specific conditions rather than universal WD fibrosis protocols. The major fibrosis-related models are summarized in **Table 5**.

**Table 5:** *In vitro* and emerging platforms for modeling copper-associated fibrogenic transition in Wilson disease.

Model status	Representative model	WD relevance	Main phenotype/readout	Main utility	Reported parameters/standardization status	Key limitations
Copper-toxicity HSC activation model (62)	CuSO4-treated LX-2 cells	Indirect WD relevance; models copper exposure to stellate cells rather than ATP7B-deficient hepatocyte injury	Increased α-SMA, collagen I, and extracellular matrix-related markers; Wnt/β-catenin involvement reported	Useful for initial testing of whether copper can directly activate fibrogenic programs in HSC-like cells	Study-specific; the 2026 HSC–LSEC study used 100 nM CuSO_4_·5H_2_O for LX-2 activation^34; this should not be generalized as a universal WD HSC concentration	LX-2 cells are immortalized; copper concentrations may not reflect physiological HSC exposure in WD liver
Copper-associated HSC–LSEC crosstalk model (34)	Copper-treated HSC–LSEC co-culture	Indirect WD relevance; models non-parenchymal cell communication under copper stress	VEGFA induction, LSEC capillarization-like changes, PDGFRβ/ERK signaling, and enhanced profibrotic communication	Useful for studying sinusoidal remodeling and vascular niche contribution to copper-associated fibrogenesis	100 nM CuSO_4_·5H_2_O was used for LX-2 activation; LX-2 and LSECs were then placed in upper/lower Transwell compartments for 24 h. A fixed LX-2:LSEC ratio was not reported in the main article	Lacks ATP7B-deficient hepatocytes and immune cells; WD specificity depends on experimental context
Proposed hepatocyte injury-to-HSC signaling model (60–62)	Copper-injured WT or ATP7B-KO hepatocytes, with conditioned-medium or co-culture extension to HSCs	WD relevance is direct at the hepatocyte level only when ATP7B is disrupted; hepatocyte-to-HSC coupling remains an emerging extension	Hepatocyte oxidative stress, autophagy, inflammatory signaling, and candidate profibrotic paracrine outputs	Useful for testing whether copper-injured hepatocytes secondarily promote HSC activation	No standardized WD-specific hepatocyte-to-HSC protocol; copper source/concentration, exposure time, conditioned-medium collection, and HSC exposure should be reported explicitly	Direct experimental validation of ATP7B-deficient hepatocyte-to-HSC signaling remains limited
ATP7B-deficient hepatocyte profibrotic model (52, 60, 61)	ATP7B-KO hepatocytes plus copper; HSC co-culture is a proposed multicellular extension	Direct at hepatocyte level; multicellular fibrosis relevance depends on co-culture design	Autophagy, ROS, inflammatory signaling, cell injury, and candidate profibrotic secretome changes	Links ATP7B loss to hepatocyte-derived candidate signals potentially capable of promoting HSC activation	For ATP7B-KO HepG2 studies, published copper paradigms range from lower/adaptive to higher/toxic exposure; Sasula et al. used 0–0.2 mmol/L as lower and 0.4–1.2 mmol/L as higher CuCl_2_ exposures, including 0.6 mmol/L for signaling analyses. These are hepatocyte-injury, not fibrosis-standardization, conditions	Without HSCs, LSECs, and macrophages, it remains an upstream injury model rather than a complete fibrosis model
Early pre-fibrotic hepatocyte stress model (52)	ATP7B-KO HepG2/C3a under low copper stress	Direct early hepatocyte model	Protein misfolding, proteostasis stress, and adaptive stress responses before overt cytotoxicity	Useful for identifying early triggers that may precede inflammation and fibrogenesis	Charbonnier et al. examined micromolar CuCl_2_ conditions (including 1, 10, and 100 μM overnight for localization and 2–200 μM for stress-response experiments); these define early hepatocyte stress, not an HSC fibrosis protocol	Does not model extracellular matrix deposition or non-parenchymal cell activation
Patient-derived hepatocyte validation model (61)	WD primary hepatocytes or WD iPSC-HLC	Direct human WD relevance	Copper-handling defects, oxidative stress, autophagy, and mutation-dependent hepatocyte stress	Useful for confirming whether mechanisms from animals and transformed cells occur in human WD backgrounds	Patient-/line-specific; no standardized WD fibrosis exposure. Report ATP7B genotype, differentiation maturity, copper condition, and culture duration	Primary cells are scarce; iPSC-HLCs are immature; fibrosis endpoints require co-culture or matrix platforms
Auxiliary human HSC model (55)	iPSC-derived hepatic stellate cells	Fibrosis-relevant auxiliary model; WD relevance requires copper exposure or WD hepatocyte-conditioned medium	HSC activation, α-SMA expression, collagen production, contractility, and matrix remodeling	More human-relevant platform than immortalized LX-2 for modeling stellate-cell activation	Protocol-dependent; report differentiation stage, baseline activation, seeding density, matrix, and duration. WD relevance requires a defined WD hepatocyte or copper context	Differentiation maturity and baseline activation vary by protocol; not WD-specific alone
Emerging 3D multicellular platform (55, 64–67)	Liver organoids, spheroids, or liver-on-chip systems with varying combinations of hepatic parenchymal and non-parenchymal compartments	Technology is WD-relevant only when an ATP7B-deficient or disease-variant hepatocyte compartment is present; current 2026 organoid/chip precedents remain non-WD-specific	Multicellular injury, inflammatory crosstalk, ECM remodeling, vascular dysfunction, and drug-response phenotypes	Promising for integrated human modeling of WD fibrogenesis and therapeutic response	No validated WD-specific standard. Report cell composition/ratio, perfusion or shear, matrix, oxygenation, copper exposure, bile-canalicular function, and longitudinal fibrosis endpoints	WD-specific versions remain underdeveloped and require standardization, validation, and reproducibility testing

## Direct copper exposure to HSCs

CuSO4-treated LX-2 cells represent a simplified model for studying the direct effects of copper on HSCs. Copper exposure can induce the expression of α-smooth muscle actin, collagen I, and other ECM-related molecules and may activate profibrotic signaling pathways such as Wnt/β-catenin signaling (62). Consequently, this model is useful for determining whether copper can directly promote HSC activation and for preliminary antifibrotic drug screening.

However, this model should not be considered a direct WD model. First, LX-2 cells are immortalized and do not fully recapitulate the quiescent-to-activated transition observed in primary HSCs. Second, the concentration and mode of copper exposure may not accurately reflect the microenvironment experienced by HSCs in WD livers. Third, the model lacks ATP7B-deficient hepatocytes, which represent the primary pathogenic driver in WD. Therefore, CuSO4-treated LX-2 cells are best classified as a copper-toxicity HSC activation model rather than a WD-specific fibrosis model (34, 55, 62).

## Hepatocyte-to-HSC injury signaling

Compared with direct copper exposure of HSCs, hepatocyte-to-HSC signaling models more closely resemble the pathological sequence underlying WD fibrosis. In WD, HSC activation is likely driven predominantly by signals arising from chronically injured hepatocytes and the altered hepatic microenvironment, although direct copper effects on HSCs may also contribute (52, 59, 61, 62). Copper-treated HepG2-conditioned medium can be used as an initial system to investigate whether injured hepatocytes release factors capable of activating HSCs. However, when wild-type HepG2 cells are used, the model primarily reflects copper-induced hepatocyte toxicity rather than ATP7B-deficiency-driven injury (59, 61, 62).

In contrast, ATP7B-KO hepatocyte-conditioned medium and ATP7B-KO hepatocyte–HSC co-culture systems possess greater disease relevance because they incorporate the underlying genetic defect responsible for impaired copper handling (52, 59, 61). A logical next-generation approach would be to combine WD patient-derived iPSC-HLCs with iPSC-derived or primary HSCs for patient-specific fibrosis modeling. Such platforms could assess how different ATP7B variants influence hepatocyte-derived profibrotic secretomes and downstream HSC activation (34, 54, 55, 62). If experimentally validated, these systems could also support evaluation of antifibrotic therapies, anti-inflammatory interventions, and the effects of ATP7B correction on fibrogenic signaling.

## HSC–LSEC crosstalk and sinusoidal remodeling

LSECs play an important role in liver fibrosis progression. Under physiological conditions, LSECs help maintain HSC quiescence and sinusoidal homeostasis. Following injury, however, LSECs may undergo capillarization-like changes that promote HSC activation and ECM deposition (34). In copper-associated liver injury, communication between HSCs and LSECs may contribute to sinusoidal remodeling through VEGFA signaling, PDGFRβ/ERK pathways, and other angiogenic and profibrotic mechanisms.

HSC–LSEC co-culture systems are therefore useful for studying non-parenchymal cell communication in response to copper exposure. These models are particularly suitable for investigating VEGFA induction, LSEC capillarization, HSC activation, and PDGFRβ/ERK signaling. In the 2026 WD-relevant study, LSECs were cultured in the lower Transwell chamber and LX-2 cells in the upper chamber for 24 h; the main article did not report a fixed LX-2:LSEC seeding ratio (34). Nevertheless, the absence of ATP7B-deficient hepatocytes and immune components limits their ability to fully recapitulate WD fibrosis. Accordingly, they should be viewed as WD-relevant sinusoidal remodeling platforms rather than comprehensive WD fibrosis models.

## Emerging 3D and liver-on-chip models

Three-dimensional organoids, spheroids, and liver-on-chip systems offer new opportunities for modeling the multicellular complexity of WD. An ideal WD liver-on-chip or organoid platform would incorporate ATP7B-deficient hepatocytes, HSCs, LSECs, Kupffer-like macrophages, ECM components, biliary-like structures, oxygen gradients, fluid shear stress, and physiologically relevant tissue architecture (55, 64, 65). Recent 2026 platforms have improved hepatocyte polarity, functional bile canaliculi, perfusion, and scalable liver-disease modeling: hESC-derived grape-clustered hepatocyte organoids demonstrated copper-responsive ATP7B translocation, and an organoid-derived microfluidic Liver-on-Chip reproduced polarized hepatic tubules and bile-canalicular transport. These are technology precedents rather than direct WD models because neither platform was established with an ATP7B-deficient WD background (66, 67).

Potential advantages of future WD-specific versions of these systems may include more realistic spatial organization of liver tissue, long-term monitoring of copper accumulation and chronic injury, simultaneous assessment of hepatocyte injury, inflammation, and fibrosis, humanized drug-response testing, and evaluation of gene therapy, mRNA therapy, and combined antifibrotic approaches. As of the literature search cutoff, direct WD-specific evidence for a stable multicellular ATP7B-deficient liver-on-chip remains limited. The immediate priority is therefore not to assume disease specificity from platform complexity, but to combine an ATP7B-deficient or patient-derived hepatocyte compartment with validated copper transport, biliary polarity, inflammatory, and fibrosis readouts.

## Translational applications: matching models to therapeutic questions

The translational value of WD models depends on how well they align with specific therapeutic questions. Rapid decoppering, conventional copper-lowering therapy, gene replacement, mutation-specific rescue, antifibrotic treatment, and personalized medicine each require distinct model systems. The overall framework is summarized in **Table 1**.

## Rapid decoppering and acute hepatic injury

Rapid decoppering strategies are primarily intended for severe copper toxicity, acute liver injury, and acute liver failure-like WD. Such applications are best evaluated in severe Atp7b-deficient rat models, including the LEC rat, because these animals exhibit substantial hepatic copper burdens, pronounced mitochondrial injury, and severe disease phenotypes. Methanobactin represents one of the most notable examples in this field. Through its extremely high copper-binding affinity, methanobactin can rapidly mobilize excess tissue copper and improve mitochondrial function and liver injury in severe copper overload settings. Severe rat models are particularly suitable for evaluating decoppering efficiency, mitochondrial protection, survival benefits, and acute safety profiles. In contrast, slowly progressing mouse models are generally better suited for studying long-term disease modification rather than acute therapeutic effects.

## Conventional decoppering and chronic disease modification

Conventional WD therapies include D-penicillamine, trientine, zinc supplementation, dietary copper restriction, and other long-term copper-regulating interventions. These approaches are most appropriately evaluated in Atp7b−/− mice, tx mice, and tx-j mice. Atp7b−/− mice allow standardized comparisons of treatment effects on hepatic copper levels, liver enzymes, steatosis, inflammation, and fibrosis. Tx mice are useful for investigating therapeutic responses under conditions of more advanced chronic copper toxicity. Because of their slower disease progression, tx-j mice are particularly suitable for studying early intervention, dietary modulation, one-carbon metabolism, and disease-modifying strategies.

Importantly, long-term studies should emphasize clinically meaningful endpoints rather than short-term reductions in liver copper alone. Relevant outcomes may include hepatic copper concentration, biliary copper excretion, urinary copper excretion, ceruloplasmin levels, histological inflammation, fibrosis severity, mitochondrial function, metabolic remodeling, and survival.

## Gene therapy and gene replacement

Gene therapy has emerged as a major therapeutic direction for WD and includes AAV-mediated ATP7B delivery, mini-ATP7B constructs, mRNA-based therapies, and genome-editing approaches. These interventions must be evaluated not only for ATP7B expression but also for proper intracellular localization, restoration of copper transport function, biliary copper excretion, durability of therapeutic effects, and immune safety. Recent preclinical work broadens this landscape beyond viral delivery: hepatocyte-targeted lipid nanoparticles carrying full-length ATP7B mRNA produced functional ATP7B expression and sustained correction of copper homeostasis in WD model mice (68).

Atp7b−/− mice are widely used as initial *in vivo* validation platforms because they allow assessment of whether restored ATP7B function, including appropriate localization and copper handling, reduces hepatic copper accumulation, improves liver function, and alleviates pathology. R780L knock-in mice provide additional opportunities to investigate therapeutic efficacy within specific mutation backgrounds. Patient-derived WD iPSC-HLCs and gene-corrected isogenic controls are valuable for evaluating ATP7B expression, protein localization, copper handling, and cellular toxicity in a human genetic context. For mutation-correction strategies, isogenic rescue in patient-derived cells provides an especially stringent causal benchmark before *in vivo* translation (63). Because AAV tropism, immune responses, and hepatocyte turnover differ substantially between mice and humans, robust development programs should include multiple validation layers: mechanistic studies in cell systems, human relevance studies in iPSC-HLCs, efficacy studies in mouse models, and, when appropriate, evaluation in large-animal or humanized platforms.

## Variant-specific rescue strategies

Variant-specific rescue approaches target ATP7B mutations that retain residual protein expression but exhibit defects in folding, stability, trafficking, or intracellular localization. Examples include pharmacological chaperones, proteostasis regulators, modulation of ER-associated degradation, trafficking rescue strategies, and agents that stabilize mutant ATP7B proteins. H1069Q cellular models and H1069Q iPSC-HLCs are particularly useful for identifying compounds that improve protein folding, reduce ER retention, restore intracellular trafficking, and recover copper transport function. R778L models can be used to investigate mutation-specific autophagy responses, necroptosis pathways, and adaptive stress mechanisms. R780L knock-in mice provide an important *in vivo* platform for testing whether candidate therapies improve copper metabolism and liver pathology. Clinical deployment, however, should not be stratified solely by variant identity unless a treatment-relevant functional defect has been demonstrated in the relevant allelic and cellular context.

Importantly, ATP7B protein abundance alone should not be considered an adequate therapeutic endpoint. Functional restoration should be demonstrated through appropriate intracellular localization, copper-responsive trafficking, restoration of vectorial/biliary copper transport or excretion, reduction of hepatic copper burden, and improvement in disease-relevant hepatic phenotypes. For precision approaches, the preferred evidence chain is variant-specific molecular defect → rescue in an isogenic or patient-derived system → restoration of copper transport → *in vivo* correction of hepatic copper handling and pathology.

## Precision-medicine use of the model ecosystem

For precision medicine, model selection should begin with the actionable defect rather than variant name alone. Misfolding/ER-retention phenotypes require systems retaining mutant protein; catalytic or copper-transport defects require functional transport readouts; gene-replacement strategies require models capable of testing hepatic delivery and biliary copper correction; and editing strategies require isogenic confirmation of on-target phenotypic rescue. Patient-derived iPSC-HLCs are most useful when linked to a matched corrected control, whereas knock-in animals are most useful when the intervention depends on the *in vivo* consequences of a specific mutant protein. This approach allows precision therapy to be mechanistically stratified without overstating clinical genotype–phenotype determinism.

## Antifibrotic and multicellular screening

The development of antifibrotic therapies for WD requires a multitiered validation strategy. Initial screening can be performed using CuSO4-treated LX-2 cells or iPSC-derived HSCs to evaluate effects on α-SMA expression, collagen I production, TGF-β signaling, and Wnt/β-catenin activation. Promising candidates should subsequently be tested in HSC–LSEC co-cultures or ATP7B-KO hepatocyte–HSC co-culture systems to determine whether they can interrupt copper-associated intercellular communication and hepatocyte-driven HSC activation. Mechanistic studies should prioritize incorporation of WD-specific hepatocyte components, including ATP7B-KO hepatocytes, WD iPSC-HLCs, or mutation-specific hepatocyte models.

Ultimately, candidate therapies should be validated *in vivo* using Atp7b−/− mice, tx mice, or LEC survivors to confirm improvements in fibrosis, inflammation, and liver function. Therefore, simplified HSC-based systems may be suitable for early-stage screening, but *in vivo* validation remains essential.

## Model selection, standardization, and reproducibility

A central principle in WD modeling is that no single experimental system can fully reproduce the entire clinical and pathological spectrum of WD. Model selection should be driven by scientific questions rather than by the pursuit of a universally superior model. **Figure 1** operationalizes this principle as a decision workflow, and **Table 1** identifies preferred versus confirmatory models when applications overlap.

## No single model recapitulates the full spectrum of WD

Each model captures specific aspects of WD pathology. LEC rats are particularly useful for severe liver injury, acute decompensation, and mitochondrial copper toxicity. Tx-j mice are suited for slow-progressing copper accumulation, steatosis, and metabolic remodeling. Atp7b−/− mice provide a standardized platform for mechanistic studies and therapeutic testing. iPSC-HLCs enable patient-specific and mutation-specific investigations. HSC-based co-cultures facilitate fibrosis research, whereas canine models offer opportunities to study long-term copper hepatopathy, dietary influences, and modifier genetics. Preference between overlapping models should be based on the endpoint that would invalidate the hypothesis if it were absent—for example, biliary copper correction for gene replacement, tissue-level ECM remodeling for antifibrotic efficacy, or acute survival/mitochondrial recovery for rapid decoppering.

Consequently, optimal study designs often involve combinations of models. Mechanistic discoveries may begin in ATP7B-KO cells, be validated in WD iPSC-HLCs, expanded to multicellular fibrosis systems, and ultimately tested in animal models. Severe-stage interventions may additionally require validation in rat models of acute copper toxicity.

## Variables affecting interpretation

Numerous variables influence WD model phenotypes and interpretation. In animal studies, genetic background, sex, age, dietary copper content, microbiome composition, and housing conditions can substantially alter copper accumulation, inflammation, fibrosis progression, and treatment responses. In cell culture systems, copper source, concentration, exposure duration, media composition, serum copper background, cell density, maturity, polarity, culture duration, and endpoint selection all affect experimental outcomes.

The transformed nature of HepG2/C3a cells, incomplete maturation of iPSC-HLCs, donor variability among primary hepatocytes, and limited lifespan of primary cultures should all be considered when interpreting findings. For fibrosis models, investigators should report whether HSCs, LSECs, Kupffer-like cells, ECM components, and ATP7B-deficient hepatocyte components are included. Without these details, observed responses may represent generic copper-related cellular reactions rather than WD-specific mechanisms.

## Reporting standards for copper injury models

To improve reproducibility and interpretability, studies of copper-related liver injury should report at minimum the following information: copper source, such as CuSO_4_, CuCl_2_, or copper complexes; copper concentration and exposure duration; culture medium composition; baseline serum copper content when applicable; intracellular copper quantification using methods such as ICP-MS or atomic absorption spectroscopy; cell viability measurements; ATP7B genotype status, including WT, KO, knock-in, patient-derived variant, or corrected control; hepatocyte maturity and polarity; fibrosis-related endpoints, such as α-SMA, collagen I, TGF-β signaling, and ECM deposition; and inclusion of non-parenchymal cells and ECM components. For co-culture studies, the cell source, seeding density or ratio, compartment configuration, timing of copper exposure relative to co-culture, and total co-culture duration should also be reported. Where a published study does not provide one of these parameters, it should be identified as unreported rather than reconstructed. These reporting standards facilitate comparisons among studies and help distinguish physiologically relevant copper stress from pathological copper overload and nonspecific toxicity.

## Future directions

Future WD liver injury models should evolve toward genotype-specific, humanized, multicellular, spatially resolved, and translationally integrated systems.

## Variant-specific and genotype-informed models

The ATP7B mutational spectrum is highly diverse, and different variants may cause distinct pathogenic mechanisms. Future efforts should focus on generating additional clinically relevant ATP7B knock-in models and integrating them with patient-derived iPSC-HLCs and isogenic correction systems. The primary objective should be a variant-function map—folding, ATPase activity, trafficking, stability, degradation, and residual copper transport—linked cautiously to clinical datasets rather than a deterministic genotype-to-progression map.

## Humanized multicellular platforms

Future *in vitro* systems should move beyond isolated hepatocyte cultures toward humanized multicellular platforms incorporating ATP7B-deficient hepatocytes, HSCs, LSECs, macrophage-like cells, ECM structures, oxygen gradients, and biomechanical cues. These platforms could simultaneously model copper accumulation, hepatocyte injury, inflammatory signaling, HSC activation, sinusoidal remodeling, and fibrosis progression. The key validation requirement is WD-specific genetic and functional fidelity, particularly copper-responsive ATP7B behavior, vectorial/biliary copper handling, and reproducible multicellular fibrosis endpoints.

## Integration of copper metallomics and spatial biology

As a copper metabolism disorder, WD research should increasingly incorporate metallomics approaches. Total hepatic copper content alone provides limited insight because copper localization, chemical state, binding partners, and cell-type-specific distribution also influence toxicity. Integrating copper metallomics with spatial transcriptomics, single-cell RNA sequencing, proteomics, metabolomics, and epigenomics could reveal dynamic cellular responses during disease progression. Such approaches may facilitate identification of early injury thresholds, fibrosis-initiating signals, treatment-response biomarkers, and novel therapeutic targets.

## Translational validation pipelines

Future WD therapeutic development should adopt tiered validation pipelines rather than relying on individual models. A rational translational workflow may include mechanistic discovery in ATP7B-KO or mutation-specific cellular systems; human validation using WD iPSC-HLCs, variant-specific iPSC-HLCs, or primary WD hepatocytes; fibrosis validation in hepatocyte–HSC/LSEC co-culture systems or multicellular platforms; *in vivo* testing in Atp7b−/− mice or knock-in models to evaluate improvements in copper metabolism and liver pathology; and severe-stage validation in LEC rats, Atp7b-defective rats, or comparative canine models for therapies targeting acute liver injury or rapid decoppering. **Figure 3** summarizes this escalation from mechanism to human relevance, tissue complexity, *in vivo* efficacy, and indication-specific confirmation.

**Figure 3:**
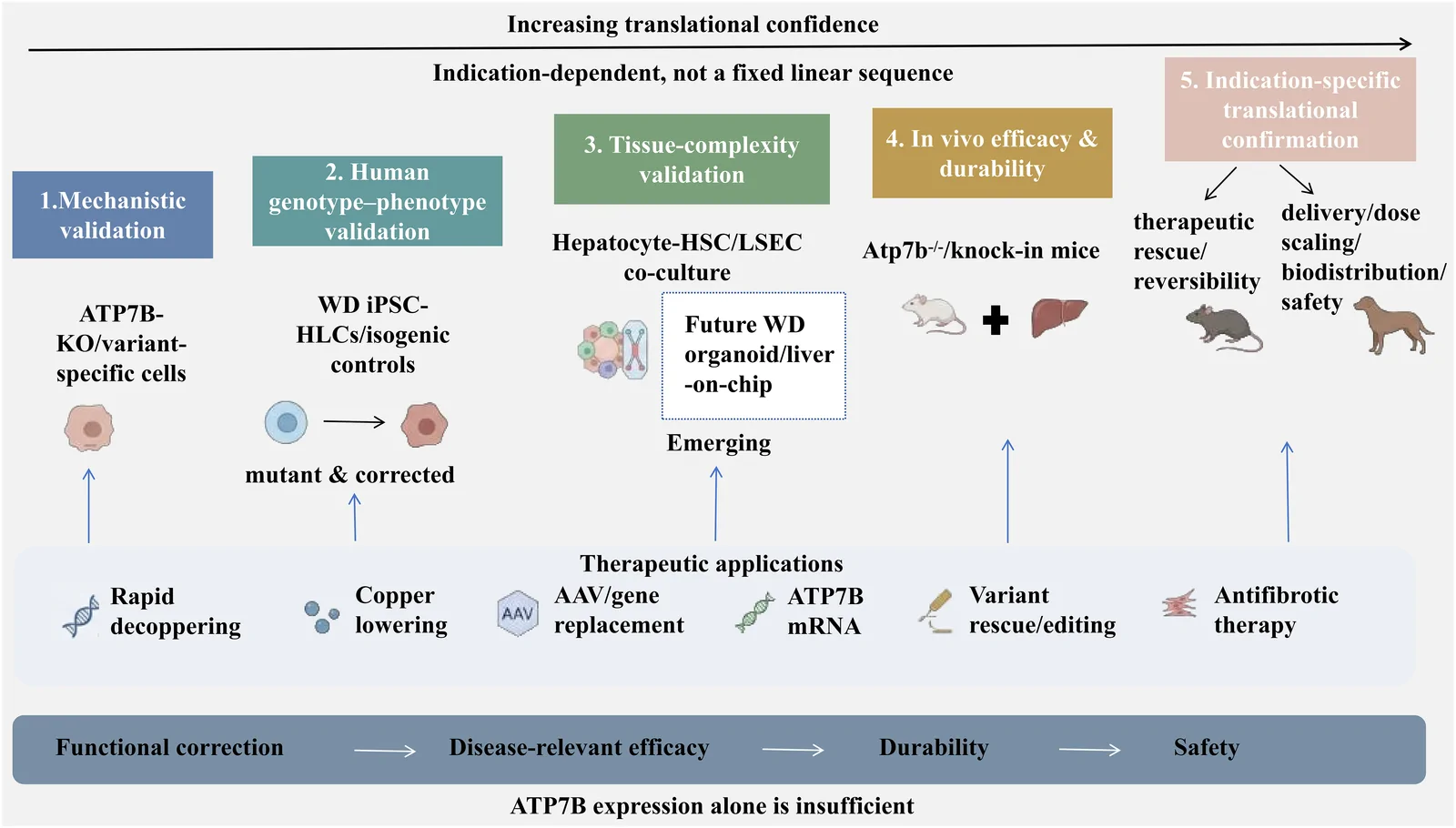
Translational validation pipeline from mechanistic discovery to therapeutic development in Wilson disease. Translational confidence is built across five complementary, indication-dependent levels. Level 1, mechanistic validation: ATP7B-KO or variant-specific cells establish target engagement and mechanistic correction. Level 2, human genotype–phenotype validation: patient-derived WD iPSC-HLCs, ideally with isogenic controls, establish human relevance. Level 3, tissue-complexity validation: multicellular co-cultures and emerging WD-specific organoid/liver-on-chip systems assess tissue-level responses. Level 4, *in vivo* efficacy and durability: Atp7b−/− or variant-specific knock-in mice establish disease-relevant efficacy and persistence. Level 5, translational confirmation: therapeutic rescue, delivery, dose scaling, biodistribution, and safety are evaluated as required by the indication. Therapeutic modalities may enter at different levels according to mechanism and intended use. Advancement should prioritize functional correction, disease-relevant efficacy, durability, and safety rather than ATP7B expression alone.

Such a multimodel validation strategy balances mechanistic depth, human relevance, tissue complexity, and translational applicability. Ultimately, the goal of WD modeling should not be to identify a universal model but to establish a flexible model ecosystem that can be tailored to specific disease stages, mechanistic hypotheses, and therapeutic objectives.

## Conclusion

WD liver injury is a dynamic pathological continuum initiated by ATP7B dysfunction and hepatic copper retention, with overlapping phases characterized by hepatocellular stress, metabolic remodeling, inflammatory amplification, fibrosis, and decompensation. Three conclusions follow from the evidence reviewed here. First, model identity should be secondary to fit-for-purpose selection: disease stage, dominant mechanism, ATP7B genetic status, and therapeutic endpoint determine whether a model is informative. Second, ATP7B-deficient models and copper-toxicity reference models answer different mechanistic questions and should not be interpreted interchangeably. Third, translational confidence increases when a finding is reproduced across complementary levels of biological complexity rather than in a single “best” model.

The principal gaps are equally clear: variant-specific experimental phenotypes remain only incompletely connected to human clinical trajectories; WD fibrosis lacks standardized multicellular parameters and validated disease-specific endpoints; and direct ATP7B-deficient multicellular organoid/liver-on-chip systems remain underdeveloped. Priority should therefore be given to isogenic human models, transparent reporting of copper and co-culture conditions, functional rather than expression-only endpoints for ATP7B rescue, and tiered validation pipelines that connect mechanistic discovery to clinically relevant therapeutic questions.
